# Systematic analysis identifies XRCC4 as a potential immunological and prognostic biomarker associated with pan-cancer

**DOI:** 10.1186/s12859-023-05165-8

**Published:** 2023-02-10

**Authors:** Yang Yu, Yanyan Sun, Zhaoxian Li, Jiang Li, Dazhi Tian

**Affiliations:** 1 Organ Transplant Center, Tianjin First Central Hospital, Nankai University, Tianjin, 300190 China; 2 School of Medicine, Nankai University, 94 Weijin Road, Tianjin, 300071 China

**Keywords:** XRCC4, Pan-cancer, Prognosis, Immune, Drug sensitivity

## Abstract

**Background:**

XRCC4 is a NHEJ factor identified recently that plays a vital role in repairing DNA double-stranded breaks. Studies have reported the associations between abnormal expression of XRCC4 and tumor susceptibility and radiosensitivity, but the potential biological mechanisms by which XRCC4 exerts effects on tumorigenesis are not fully understood. This study aimed to systematically investigate the role of XRCC4 across cancer types.

**Methods:**

The TIMER, GTEX and Xiantao Academic database were used to interpret the expression of XRCC4. Genomic alterations and protein expression in human organic and tumor tissues were applied in cBioPortal and the Human Protein Atlas databases. Correlations between XRCC4 expression and immune and molecular subtypes were analyzed by using the TISIDB database. Protein–protein interactions, GO and KEGG enrichment were also applied for XRCC4-related genes. The TIMER and the Tumor Immune Single Cell Hub (TISCH) online databases were used to explore the relationship between XRCC4 and tumor immune microenvironment. Drug sensitivity information was acquired from the CellMiner database to analyze the effect of XRCC4 on sensitivity analysis.

**Results:**

The XRCC4 expression was significantly upregulated in 15 tumor types and downregulated in two tumor types compared with the normal tissues, most of which were validated by the results of Xiantao academic platform. XRCC4 was expressed at intermediate level in malignant cells. The XRCC4 expression was related to the molecular and immune subtypes of human cancers, and the survival outcome of 11 types of cancers, including KIRC, STAD and LIHC. The main type of frequent genetic alteration is amplification. Strong correlations were also found between XRCC4 and immune checkpoint genes in 33 human cancers. Furthermore, the abnormal expression of XRCC4 was related to immune cell infiltration and drug sensitivity. Enrichment analysis showed that XRCC4 was significantly correlated with DNA damage response.

**Conclusions:**

This comprehensive pan-cancer analysis suggested that XRCC4 may play a vital role in the prognosis and immunotherapy response in cancer patients, and it is a promising therapy target in the future.

**Supplementary information:**

The online version contains supplementary material available at 10.1186/s12859-023-05165-8.

## Introduction

Cancer remains a main public health challenge to the improvement of quality and length of life. According to the 2018 global cancer statistics, 18.1 million were newly diagnosed with cancer, and 9.6 million deaths due to cancer globally [1]. As our understanding of the molecular basis of tumorigenesis has improved with advances in early diagnosis and treatment, deaths from cancers declined significantly between 1991 and 2017[2]. Today, cancer immunotherapy and targeted therapy have become prominent cancer treatment, especially immune checkpoint blocking therapy, but cures remain rare. Therefore, it raises an urgent necessary for the development of reliable diagnostic and prognostic biomarkers and novel therapeutic strategies. With the improvement of the public databases, bioinformatics analysis was applied to discover potential therapeutic targets by performing pan-cancer expression analysis [3, 4].

Genomic instability is an enabling characteristic of cancer which promotes the malignant transformation [5].DNA damage response (DDR) is essential to protect cells maintain genome integrity by against some acquired genome changes and monitor exogenous or endogenous DNA damage [6]. X-ray repair cross complementing protein 4 (XRCC4) is recognized as the main player involved in repairing DNA double-stranded breaks by interacting with Ku70/ Ku80 complex and as a crucial scaffold protein between this complex and DNA ligase IV, which involves in the final step of non-homologous end-joining (NHEJ) [7–9]. Studies have found XRCC4 expression in various tumors, including uterine cervical cancer [10], breast cancer [11], esophageal cancer [12] and hepatocellular carcinoma [13]. In addition, Takada et al. found that patients with high XRCC4 and Ku86 expression uterine cervical cancer had a significantly higher rate of distant metastasis [10]. As XRCC4 involved in the DDR, it has the potential to be a biomarker for the sensitivity of tumor cells to chemotherapy and radiotherapy [10, 12, 14, 15]. Besides that, XRCC4 has been proven to be negatively related to the overall survival (OS) and tumor recurrence-free survival (RFS) in hepatocarcinoma [16, 17]. However, the mechanisms of molecular biology and clinical implications of XRCC4 in cancers are still unclear and need to be further elucidated.

Until now no study has focused on the association between XRCC4 and pan-cancer. Considering the specific role of XRCC4 in cancers, we conducted the systemic analysis to explore the potential role of XRCC4 across cancer types. Transcriptomic dataset available via TCGA and GTEx to explore expression profile of XRCC4 across cancer types and normal tissues. Meanwhile, we considered genetic alteration, prognostic value of XRCC4, and potential association between the level of expression of XRCC4 and the clinical features and infiltrating immune cells across cancer types. Then, we applied enrichment Analysis to elucidate the biological function. Furthermore, the CellMiner™ database was used to carry out drug sensitivity analysis of XRCC4.

## Materials and methods

### Data acquisition and gene expression analysis

TIMER2 (tumor immune estimation resource, version 2) web (http://timer.cistrome.org/) was used to explore the expression difference of XRCC4 between tumor types and corresponding normal tissues of the TCGA database. For the tumors with highly limited or without normal tissues [e.g., TCGA-THYM (Thymoma), TCGA-LGG (Brain lower grade glioma), etc.], the GEPIA2 (Gene Expression Profiling Interactive Analysis, version 2) web (http://gepia2.cancer-pku.cn/#analysis) [18] was used to generate box plots of expression of tumor tissues and adjacent normal tissues of the GTEx (Genotype-Tissue Expression) database, under the settings of *P*-value cutoff = 0.01, log_2_FC (fold change) cutoff = 1, and “Match TCGA normal and GTEx data”. The XIANTAO Academic (https://www.xiantao.love/products) was used for the paired comparison of XRCC4 expression of the pan-cancer with case-matched adjacent normal tissues from the TCGA project. Cancer types with sample size less than 15 were excluded. The TISCH (http://tisch.comp-genomics.org) database [19] was used to systematically investigate the cell composition of the tumor tissue and their contributions to the expression of XRCC4.

### Immunohistochemistry (IHC) staining

We obtained IHC images of XRCC4 protein expression in four tumors tissues and normal tissues in healthy control from the HPA (the Human Protein Atlas) (http://www.proteinatlas.org/), including liver hepatocellular carcinoma (LIHC), breast cancer (BRCA), lung Adenocarcinoma (LUAD), and Renal-cancer to explore the protein expression differences.

### Survival prognosis analysis

We obtained the OS and DFS (disease-free survival) from GEPIA2 platform, and RFS (recurrence-free survival) from the Kaplan–Meier Plotter platform (www.kmplot.com) to explore the prognostic value of XRCC4 in human cancers across all TCGA tumors. [20, 21]. The median XRCC4 expression was used as the threshold to classify the high-expression and low-expression subgroups [18]. Log-rank test was applied and hazard ratios (HRs) and corresponding 95% confidence intervals (CIs) were calculated, with the significant level set at *P* < 0.05.

### Genetic alteration analysis

The cBioPortal database (https://www.cbioportal.org/) was used to explore the genetic alteration features of XRCC4 in cancer tissues. We collect the genetic alteration characteristics of XRCC4 across all TCGA tumors [22], including the alteration frequency, mutation type, multiple alterations, structure variation and deep deletion.

### Correlations between XRCC4 expression and immune and molecular subtypes

We investigated the correlations between XRCC4 expression levels and immune or molecular subtypes of cancer types by using the TISIDB database (http://cis.hku.hk/TISIDB/index.php), which is an online integrated abundant human cancer datasets [23]. Differences were considered statistically significant when *P* < 0.05.

### Correlations between XRCC4 expression and immune checkpoint genes, immune infiltration cells and marker genes of the immune cells

The SangerBox website (http://sangerbox.com/Tool) is an online platform for data analysis based on TCGA. We used it to analyze the relationships between XRCC4 expression and immune checkpoint (ICP) genes. The TIMER database was used to explore the correlations between XRCC4 expression and six immune cells (CD4^+^ T cells, CD8^+^ T cells, B cells, neutrophils, macrophages, and dendritic cells) in the tumor microenvironment of human cancers. Then, we explored the correlation between XRCC4 expression and marker genes of the main immune cells using the TIMER database, including CD8 + T cells, T cells (general), B cells, Monocyte cells, tumor-associated macrophages (TAMs), M1 Macrophages, M2 Macrophages, Neutrophils, Natural killer cells, and dendritic cells [24, 25]. The statistical tests were two-sided with an alpha of 0.05.

### XRCC4 associated protein–protein interaction network and gene enrichment analysis

The STRING website (https://string-db.org/) was used to acquire the top 50 XRCC4-binding proteins. We set the following main parameters: minimum required interaction score [“Low confidence (0.150)”], meaning of network edges (“evidence”), max number of interactors to show (“no more than 50 interactors” in 1st shell) and active interaction sources (“Experiments, Text mining, Databases”). Visualization of protein–protein interaction (PPI) network was realized by Cytoscape (version 3.9.0). Next, we obtained the top 100 XRCC4 expression-related genes via the GEPIA2 database, and conducted the Gene Ontology (GO) and Kyoto Encyclopedia of Genes and Genomes (KEGG) [26] enrichment analyses for the top 50 XRCC4 binding proteins via the XIANTAO Academic (https://www.xiantao.love/products).

### Drug sensitivity analysis

We downloaded NCI-60 compound activity data and corresponding RNA-seq expression dataset from the CellMiner™ (https://discover.nci.nih.gov/cellminer/home.do). The “limma”,“ggplot2”, and “ggpubr” packages of R (version 4.1.2) were performed. Except that, we also collected the IC50 of small molecules in cell lines and its corresponding mRNA gene expression from Genomics of Drug Sensitivity in Cancer (GDSC) and Genomics of Therapeutics Response Portal (CTRP) to explore the potential correlations between XRCC4 expression and drug sensitivity.

### Statistical analysis

Wilcoxon rank-sum test and Spearman rank test were carried out to compare the XRCC4 expression differences and correlation between tumor and normal tissues, respectively. Paired *t*-test was performed to evaluate the differences between tumor and adjacent normal tissues. Univariate Cox regression analysis and the log-rank test were applied to evaluate the prognostic role of XRCC4 expression in each cancer. Spearman correlation analysis was performed to evaluate the relationships between XRCC4 and other factors, such as immune cell infiltration levels and immune related genes (immune checkpoints and markers of immune infiltrations). The Benjamini–Hochberg (BH) false discovery rate (FDR) method was used to account for multiple testing, and results with *P* < 0.05 and *FDR* < 0.10 were considered statistically significant.

## Results

### XRCC4 expression across cancer types and normal tissues

**Fig. 1:**
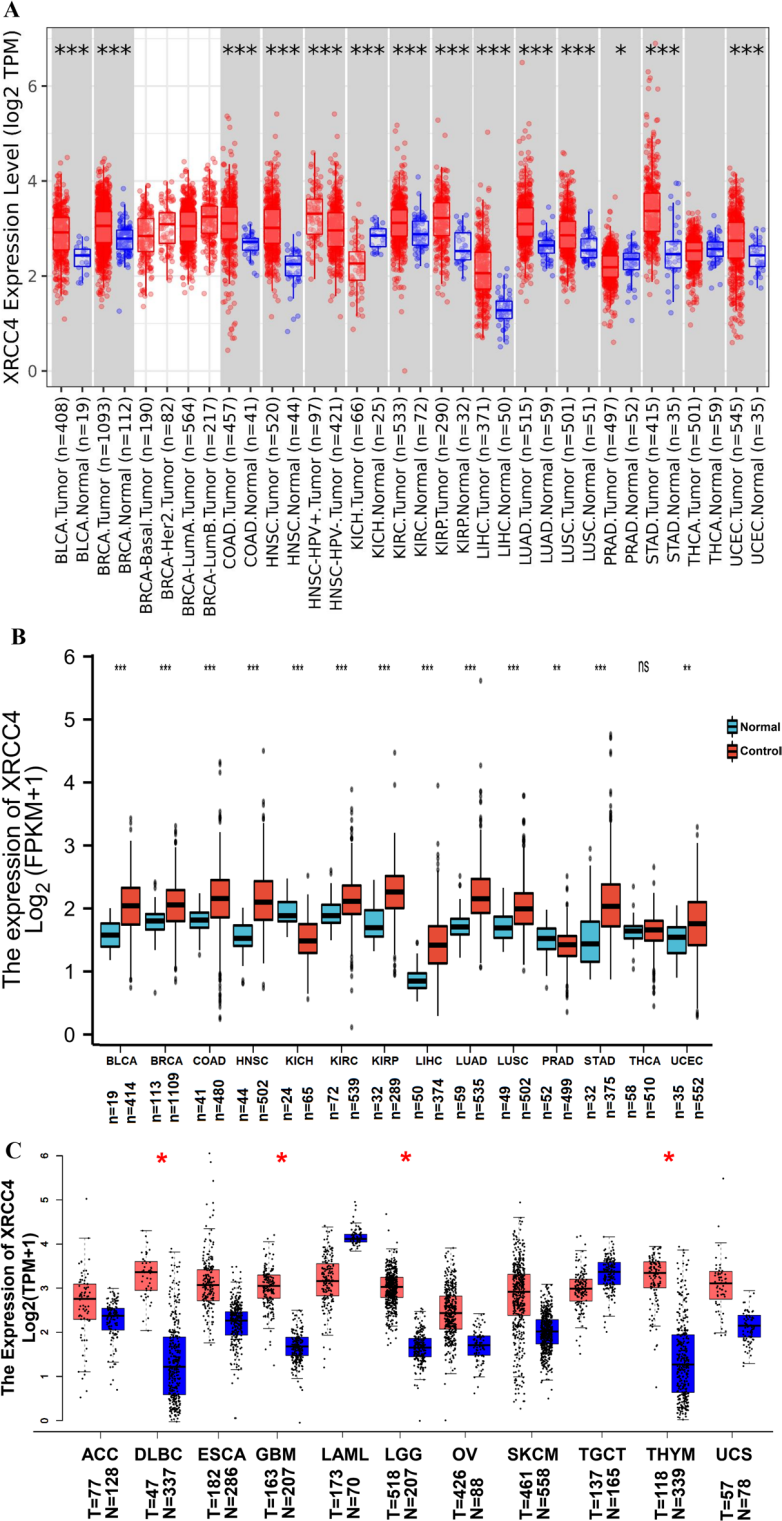
XRCC4 expression in human cancers. **A** XRCC4 expression in TCGA tumors and adjacent normal tissues in different cancer types from the TCGA database analyzed by the TIMER database. (*P < 0.05, ** *P *< 0.01, ***P< 0.001). **B** XRCC4 expression in TCGA tumors and adjacent normal tissues analyzed by the Xiantao Academic; **C** XRCC4 expression in several cancers and paired normal tissue in the GEPIA database. (*P < 0.05, **P < 0.01, ***P < 0.001)

We explored XRCC4 expression in tumor and normal tissues from the TIMER database and found that XRCC4 was significantly upregulated in 11 cancer types, including, bladder urothelial carcinoma (BLCA), breast invasive carcinoma (BRCA), colon adenocarcinoma (COAD), head and neck squamous cell carcinoma (HNSC), kidney renal papillary cell carcinoma (KIRC), kidney renal papillary carcinoma (KIRP), liver hepatocellular carcinoma (LIHC), lung adenocarcinoma (LUAD), lung squamous cell carcinoma (LUSC), stomach adenocarcinoma (STAD) and uterine corpus endometrial carcinoma (UCEC) than normal tissues. However, XRCC4 was downregulated only in kidney chromophobe (KICH) and prostate adenocarcinoma (PRAD) (Fig. 1A). The result of comparison of XRCC4 expression of the pan-cancer with normal tissues in the XIANTAO Academic showed that XRCC4 mRNA expression was significantly high among most cancer types except KICH and PRAD, which was consistent with the TIMER database results (Fig. 1B). We further analyze the cancers without normal tissues in the TIMER database via the GEPIA database, and the results showed that XRCC4 was significantly upregulated in lymphoid neoplasm diffuse large B-cell lymphoma (DLBC), glioblastoma (GBM), LGG and THYM (Fig. 1C).

We used six datasets (BRCA_GSE176078, KICH_GSE159115, KIRC_GSE171306, LIHC_GSE146409, PRAD_GSE141445 and STAD_GSE134520) of the TISCH database to evaluate XRCC4 expressions in the tumor microenvironment related immune cells. We found that different cancer types vary in the cells component and the proportion of tumors. As to the major lineage, for instance, malignant, fibroblast, endothelial, DC, CD8Tex, CD4Tconv, B cell, Tprolif, SMC, Plasma, and Mono/Macro the of BRCA_GSE176078, endothelial, epithelial, fibroblast, hepatocyte, malignant and Mono/Macro the of LIHC_GSE146409. XRCC4 was expressed at intermediate level in malignant cells among the datasets (Additional file 1), and XRCC4 expression level was quite different in distinct cell types, which might be the source of cancer microenvironment heterogeneity.

**Fig. 2:**
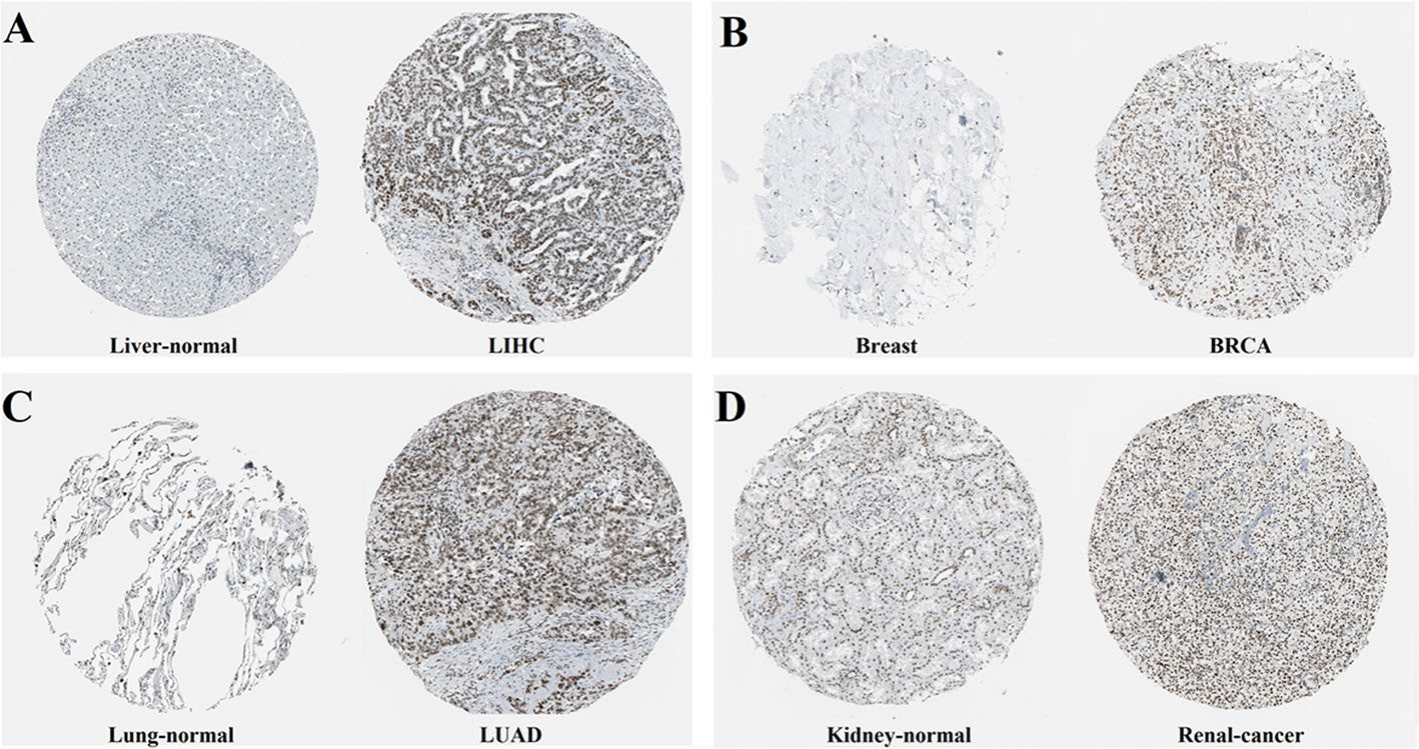
Protein expression level of XRCC4 in several cancers and normal tissues. **A** Liver, **B** Breast, **C** Lung and (D) Kidney, × 100

We further explored the IHC results in HPA database to figure out XRCC4 expression in tumors. The result showed that normal liver, breast, lung and renal had negative or medium XRCC4 IHC staining, but tumor tissues showed medium or strong IHC staining. (Fig. 2).

### Prognostic value of XRCC4 across cancer types

**Fig. 3:**
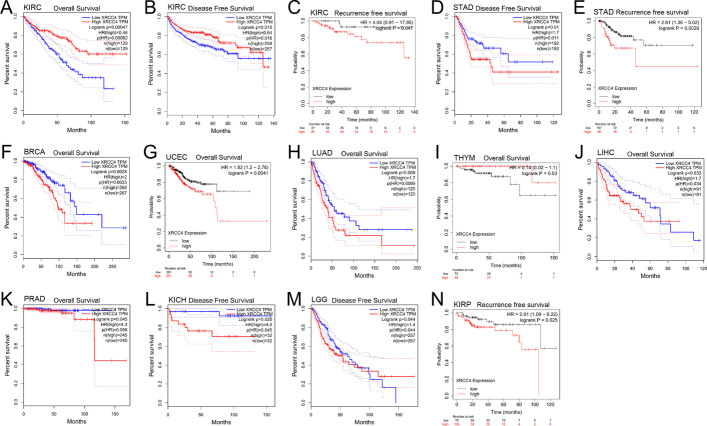
Survival analysis with high and low YTHDF1 expression. **A–C** Kaplan–Meier analysis of the association between XRCC4 expression and OS, DFS and RFS in KIRC analyzed by the GEPAI database and the Kaplan–Meier plotter database, **D, E** Kaplan–Meier analysis of the association between XRCC4 expression and DFS **(D)** and RFS **(E)** in STAD analyzed by the GEPAI database and the Kaplan–Meier plotter database, respectively, **F–K** Kaplan–Meier analysis of the association between XRCC4 expression and OS in BRCA **(F),** UCEC **(G),** LUAD **(H),** THYM **(I),** LIHC **(J),** and PRAD **(K)**; **L, M** Kaplan–Meier analysis of the association between XRCC4 expression and DFS in KICH **(L)** and LGG **(M)**; **O, P** Kaplan–Meier analysis of the association between XRCC4 expression and RFS in KIRP **(L)** and HNSC **(O)**; The red line represents high XRCC4 expression and the blue line represents low XRCC4 expression. OS, overall survival; PFI, progression-free interval; DSS, disease-specific survival

To further investigate the potential prognostic value of XRCC4 in cancers, univariate Cox regression and log-rank test were carried out. Figure 3 presented the expression of XRCC4 was significantly negative associated with OS for cancers, including UCEC (HR = 1.82, *P* = 0.0041), LUAD (HR = 1.70, *P* = 0.0090), LIHC (HR = 1.70, *P* = 0.0330), PRAD (HR = 4.30, *P* = 0.0450), and BRCA (HR = 2.0, *P* = 0.0028). We found the low expression of XRCC4 was positive associated with OS for THYM (HR = 0.14, *P* = 0.0300) and KIRC (HR = 0.46, *P* = 0.0005). The high expression of XRCC4 was positive associated with poor DFS in KICH (HR = 4.90, *P* = 0.0260), STAD (HR = 2.61, *P* = 0.0100), and LGG (HR = 1.40, *P* = 0.0440), but with favorable DFS in KIRC (HR = 0.64, *P* = 0.0150). The result also indicated a negative correlation between XRCC4 overexpression and favorable RFS in patients with KIRC (HR = 4.04, *P* = 0.0470), KIRP (HR = 2.61, *P* = 0.0250) and STAD (HR = 2.61, *P* = 0.0029).

### Genetic alteration of XRCC4 across cancer types

**Fig. 4:**
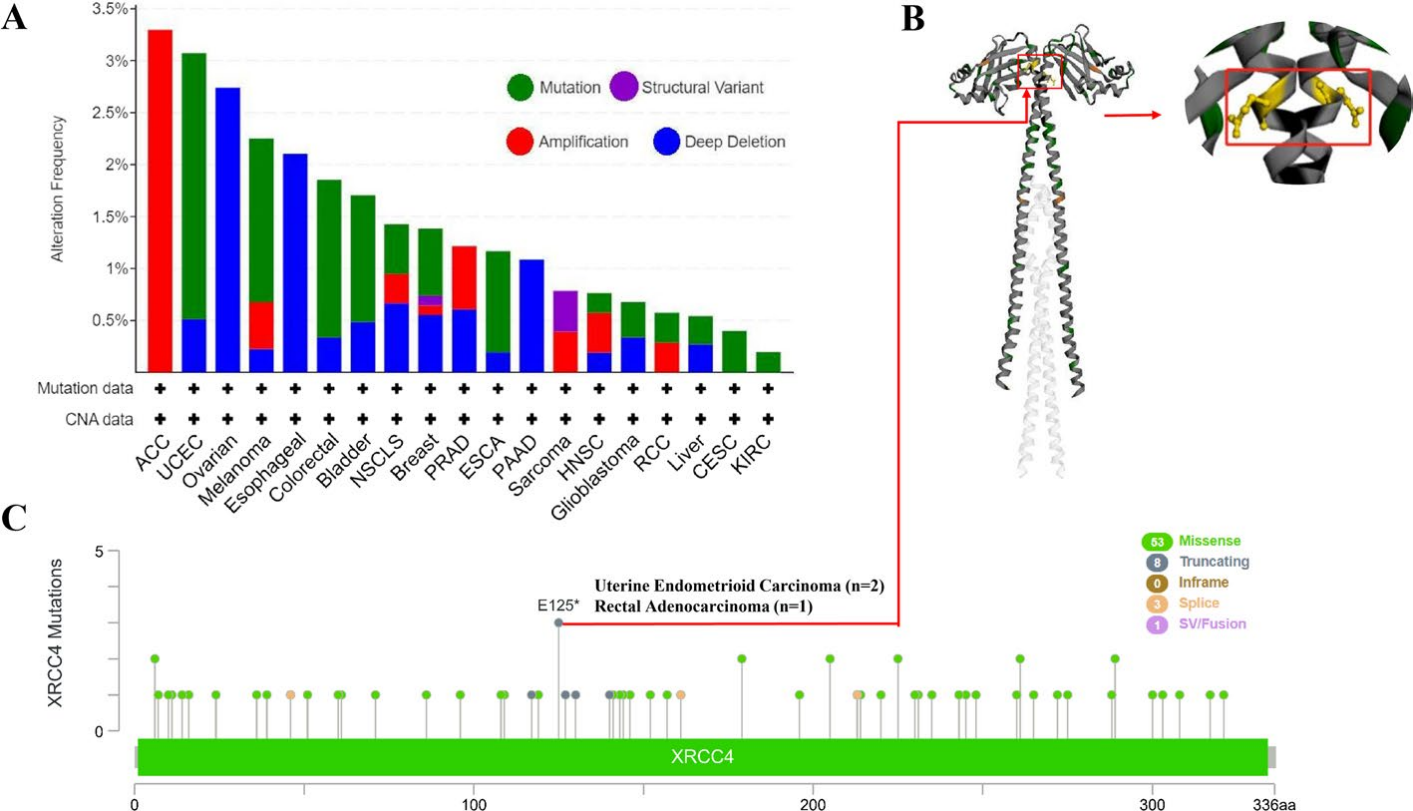
The genetic alterations of XRCC4 across cancer types analyzed by the cBioPortal database. **A** Alterations summary of XRCC4 in TCGA pan-cancer datasets; **B** 3D protein structure of XRCC4; **C** The mutation types, number and sites of the XRCC4 genetic alterations

Due to the abnormal expression of XRCC4 across cancers, we explore the genetic alteration of XRCC4 in human cancers. According to the analysis result, the highest frequency of XRCC4 alteration appeared for pan-cancer patients with adrenocortical carcinoma (ACC), endometrial cancer, ovarian and melanoma (> 2%) using the cBioPortal database. Amplification, deep deletion and mutation are the main type of frequent genetic alterations of XRCC4. (Fig. 4A). In Fig. 4B, we showed additional genetic alterations and their locations within XRCC4. As shown in Fig. 4C, we acquired the 3D structure of XRCC4 protein.

### XRCC4 is correlated with molecular and immune subtypes in cancers

**Fig. 5:**
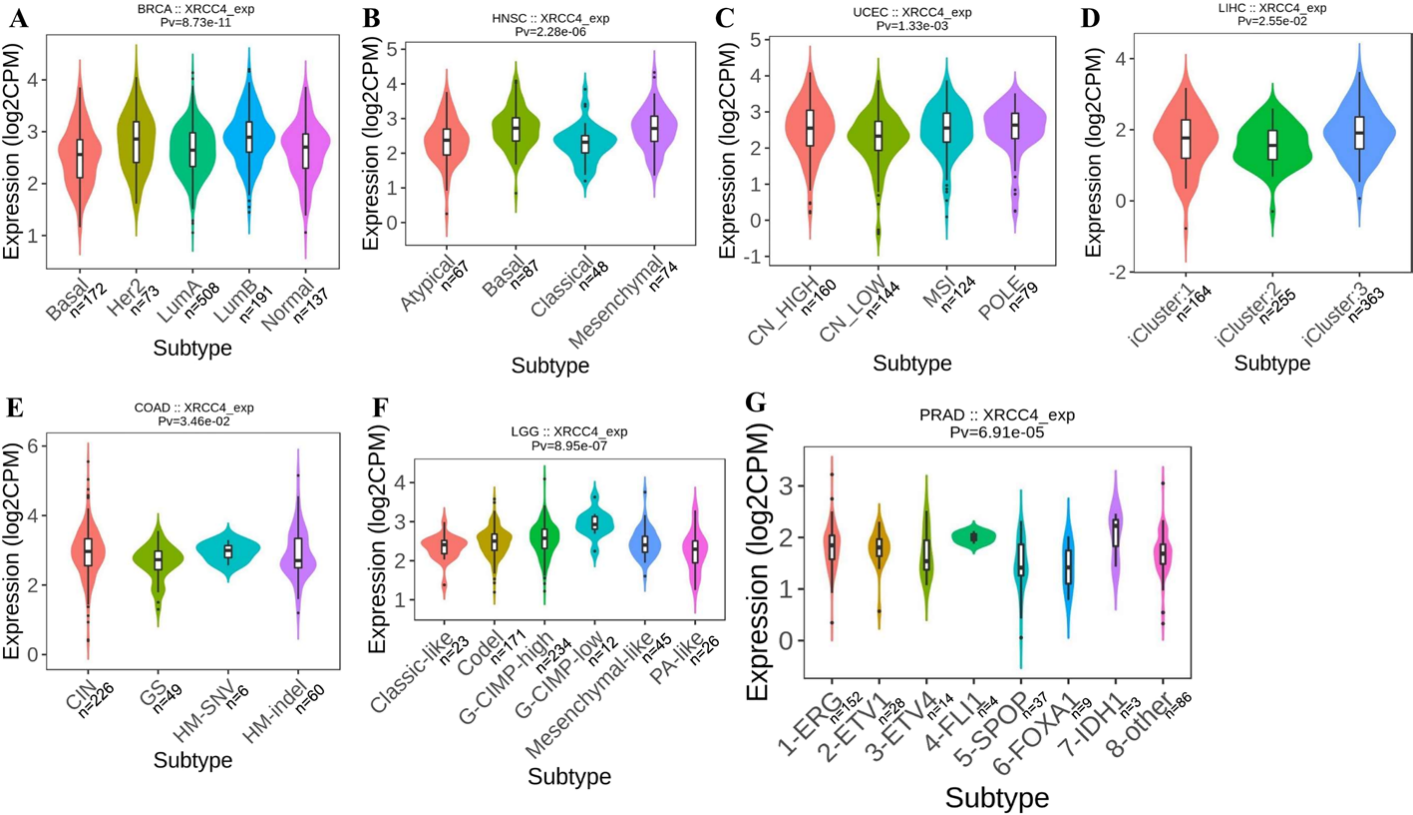
The relationship between XRCC4 expression and pan-cancer molecular subtypes. **A** in BRCA, **B** in HNSC, **C** in UCEC, **D** in LIHC, **E** in COAD, **F** in LGG and **G** in PRAD

**Fig. 6:**
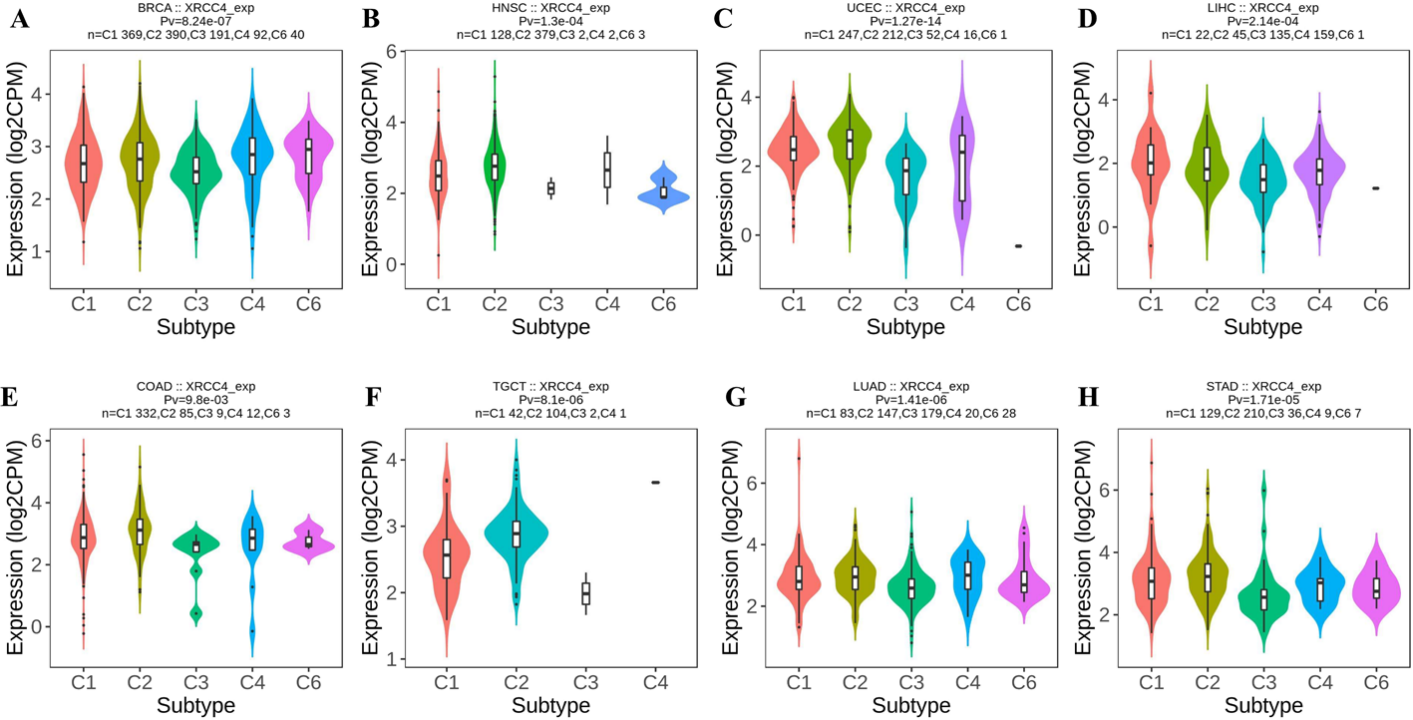
The relationship between XRCC4 expression and pan-cancer immune subtypes. **A** in BRCA, **B** in HNSC, **C** in UCEC, **D** in LIHC, **E** in COAD, **F** in TGCT, **G** in LUAD and **H** in STAD

The correlations between XRCC4 expression and molecular and immune subtypes of human cancers were explored by TISIDB database. The results showed that XRCC4 expression was significantly correlated with different molecular subtypes in BRCA (*P* < 0.0001), HNSC (*P* < 0.0001), UCEC (*P* = 0.0013), LIHC (*P* = 0.0255), COAD (*P* = 0.0346), LGG (*P* < 0.0001) and PRAD (*P* < 0.0001) (Fig. 5). The immune subtypes were divided into categories including C1 (wound healing), C2 (IFN-γ dominant), C3 (inflammatory), C4 (lymphocyte depleted), C5 (immunologically quiet) and C6 (TGF-β dominant).The correlations between XRCC4 expression and immune subtypes in BRCA (*P* < 0.0001), HNSC (*P* = 0.0001), UCEC (*P* < 0.0001), LIHC (*P* = 0.0002), COAD (*P* = 0.0098), TGCT (*P* < 0.0001), LUAD (*P* < 0.0001) and STAD (*P* < 0.0001) were shown in Fig. 6. We also found that XRCC4 expression was related to immune subtypes in BLCA, SKCM, KIRP, THCA, KIRC, OV, LUSC, SARC, READ and PCPG, which were shown in Additional file 2.

### XRCC4 is correlated with immune checkpoint genes in cancers

**Fig. 7:**
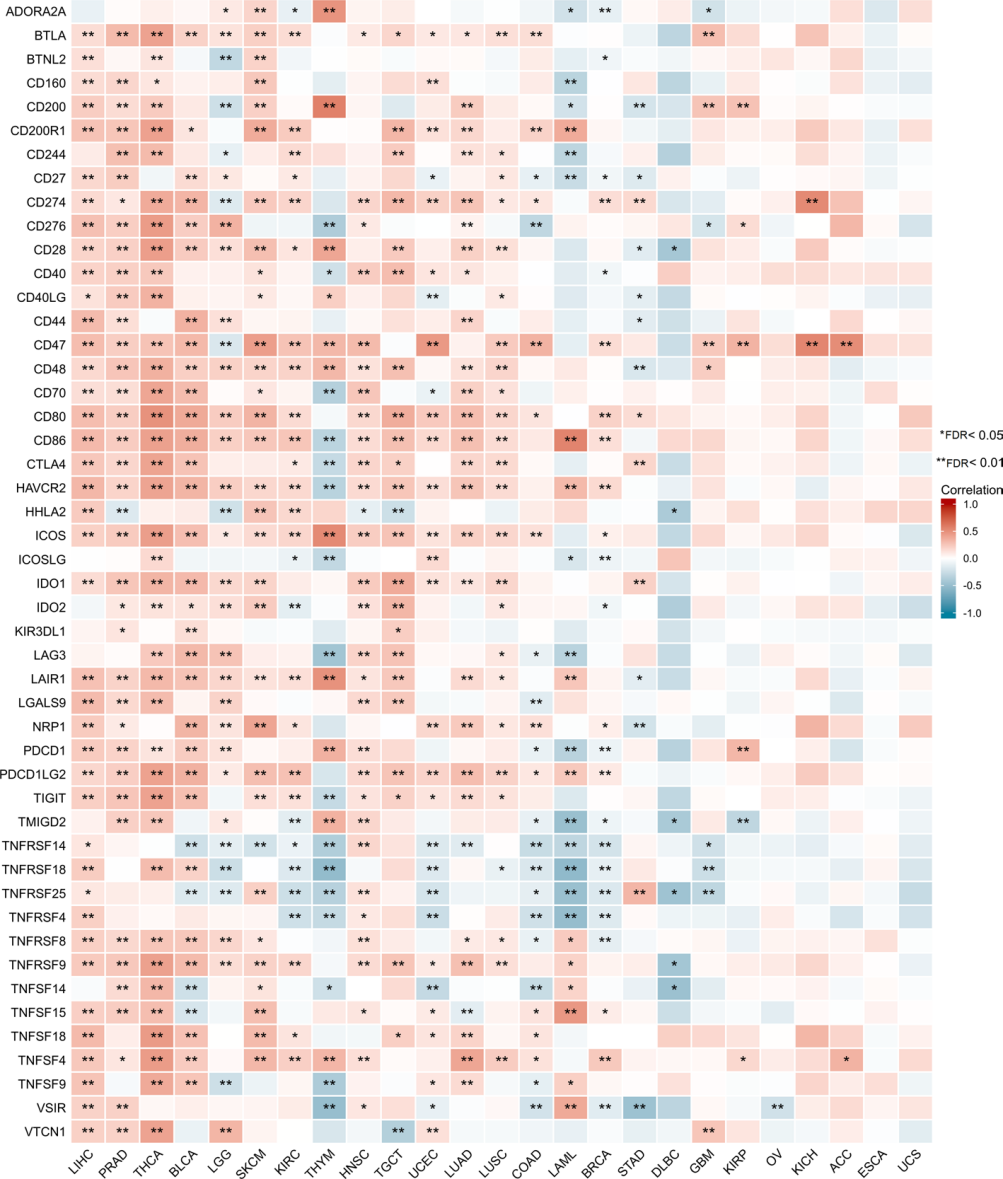
The relationship between XRCC4 expression and pan-cancer immune checkpoint genes

Figure 7 summarized the correlations between XRCC4 expression and immune checkpoint genes across cancer types. Among the 48 immune checkpoint genes, strong correlations (≥ 5 genes) with XRCC4 expression were found in 33 human cancers, including LIHC, PRAD, SARC, THCA, BLCA, LGG, SKCM, UVM, KIRC, THYM, HNSC, TGCT, UCEC, LUAD, LUSC, PCPG, COAD, LAML, BRCA, STAD, DLBC, PAAD, READ, GBM, KIRP, CESC, OV and KICH (FDR < 0.1). It is noteworthy that XRCC4 expression was associated with 41 of 48 immune checkpoint genes in LIHC, in which 40 of the 41 were positively related to immune checkpoint genes. We also found that XRCC4 expression and 15 of 48 immune checkpoint genes were all negatively correlated in DLBC. Detailed information was shown in Additional file 4: Table s1.

### XRCC4 is correlated with immune cell infiltration and marker genes of immune cells

**Fig. 8:**
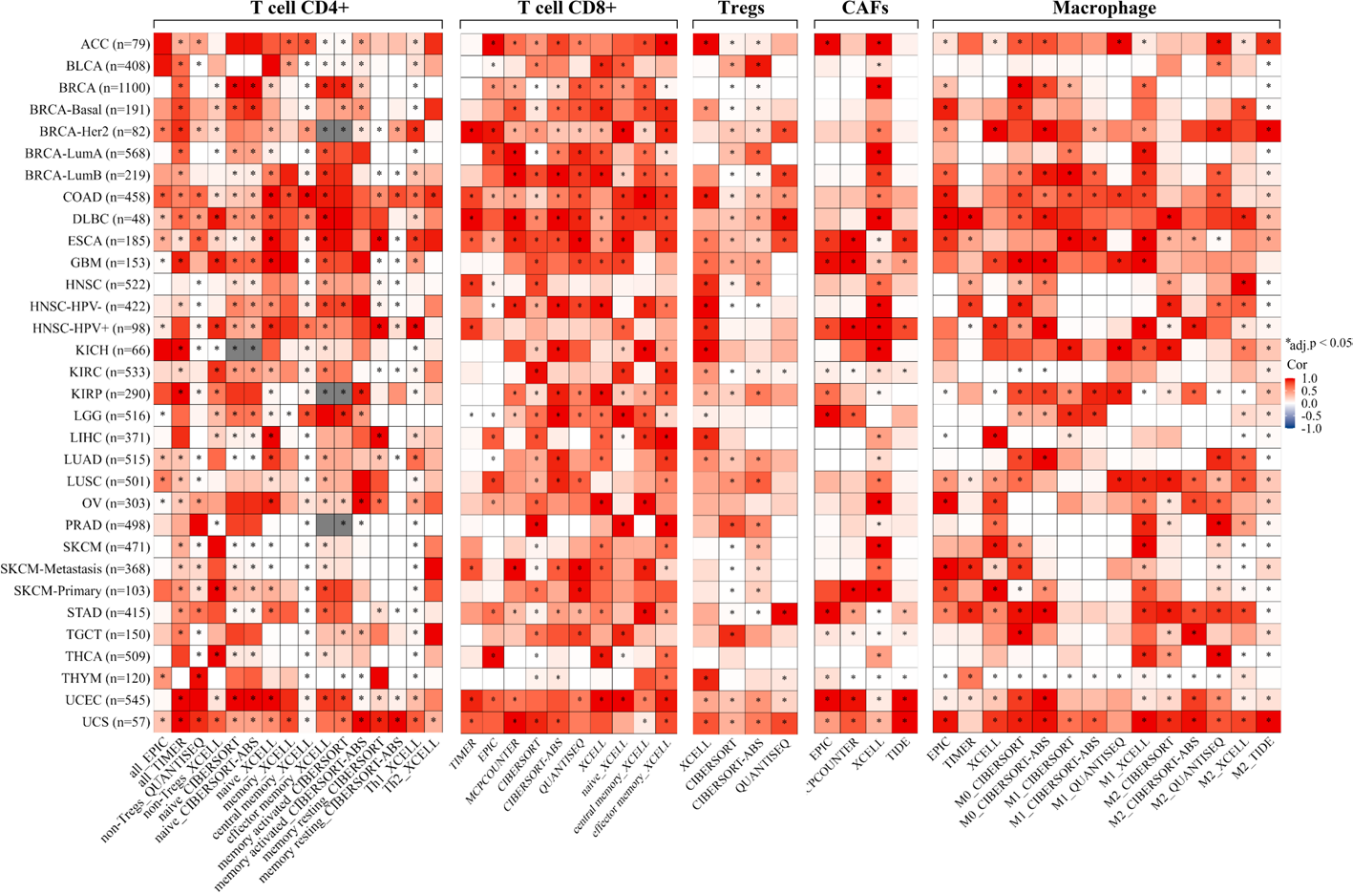
Correlation heatmaps using different algorithms showing the relationship between XRCC4 expression and infiltrating of CD4 + , CD8 + , regulatory T cells, CAFs, as well as macrophage across all types of cancer in TCGA, analyzed by TIMER2

As immune cell infiltration in the TME was strongly correlated with the effects of the immunotherapy, we further analyzed the correlation between XRCC4 expression and immune cell infiltration in human pan-cancer. We found varying directions and strengths of correlations between XRCC4 expression and immune cell infiltration. XRCC4 expression was significantly correlated with infiltrating levels of memory CD4 + T cells in 19 types of cancers, Th1 CD4 + T cells in 12 types of cancers, Th2 CD4 + T cells in 20 types of cancers, CD8 + T cells in 15 types of cancers by TIMER, cancer associated fibroblast in 13 types of cancers by TIDE, M1 macrophages in 12 types of cancers by QUANTISEQ and M2 macrophages in 14 types of cancers by TIDE. It is worth noting that the correlations between XRCC4 expression and infiltrating levels of M1 macrophages in BLCA, HNSC and THYM were consistent regardless of the algorithm used, reflecting these associations are stable (Fig. 8, Additional file 3). The detailed results and adjusted *P* value were shown in Additional file 5: Table s2.

Then, we further investigated the associations between XRCC4 expression and markers of immune cells in human pan-cancer. We found that XRCC4 expression was strongly associated with marker genes of Th1 cells, DC, TAM, M2 macrophage, monocyte, regulatory T cells (Tregs) and Th2 cells across cancer types. For example, XRCC4 expression had a strong association with STAT1 in Th1 cells, CD86 in monocyte, NRP1 in DC, IL10 in TAM, MS4A4A in M2 macrophage, STAT5B in Tregs, STAT6 in Th2 in most of cancer types, detailed information was shown in Additional file 6: Table s3. In the main text, we took PRAD and THCA for instance to show the underlying immune function of XRCC4. As shown in Table 1, XRCC4 expression was strongly correlated with more than 80% included marker genes in PRAD and THCA.

**Table 1:** Correlation between XRCC4 and relate genes and markers of immune cells analyzed by TIMER

Description	Gene markers	PRAD			THCA		
		Cor	*P*	adj.*p*	Cor	*P*	adj.*p*
CD8 + T cell	CD8A	0.308	***	***	0.127	0.004	0.017
	CD8B	0.320	***	***	0.219	***	***
T cell (general)	CD3D	0.297	***	***	0.210	***	***
	CD3E	0.302	***	***	0.205	***	***
	CD2	0.334	***	***	0.231	***	***
B cell	CD19	0.242	***	***	0.175	***	0.002
	CD79A	0.251	***	***	0.196	***	***
Monocyte	CD86	0.319	***	***	0.315	***	***
	CD115 (CSF1R)	0.325	***	***	0.259	***	***
TAM	CCL2	0.204	***	***	0.260	***	***
	CD68	0.239	***	***	0.324	***	***
	IL10	0.246	***	***	0.279	***	***
M1 Macrophage	INOS (NOS2)	0.198	***	***	0.190	***	***
	IRF5	0.192	***	***	0.354	***	***
	COX2(PTGS2)	0.318	***	***	0.378	***	***
M2 Macrophage	CD163	0.260	***	***	0.315	***	***
	VSIG4	0.262	***	***	0.271	***	***
	MS4A4A	0.234	***	***	0.303	***	***
Neutrophils	CD66b (CEACAM8)	0.019	0.667	0.813	0.232	***	***
	CD11b (ITGAM)	0.349	***	***	0.281	***	***
	CCR7	0.303	***	***	0.259	***	***
Natural killer cell	KIR2DL1	0.030	0.499	0.798	− 0.056	0.205	0.548
	KIR2DL3	0.033	0.461	0.753	− 0.009	0.836	0.904
	KIR2DL4	0.185	***	***	− 0.034	0.442	0.630
	KIR3DL1	0.122	0.007	0.115	− 0.004	0.933	0.956
	KIR3DL2	0.167	***	0.004	0.077	0.081	0.360
	KIR3DL3	-0.028	0.529	0.705	− 0.036	0.413	0.705
	KIR2DS4	0.083	0.065	0.434	− 0.029	0.520	0.774
Dendritic cell	HLA-DPB1	0.284	***	***	0.244	***	***
	HLA-DQB1	0.250	***	***	0.157	***	0.004
	HLA-DRA	0.349	***	***	0.299	***	***
	HLA-DPA1	0.335	***	***	0.255	***	***
	BDCA-1(CD1C)	0.402	***	***	0.366	***	***
	BDCA-4(NRP1)	0.216	***	***	0.170	***	***
	CD11c (ITGAX)	0.211	***	***	0.231	***	***
Th1	T-bet (TBX21)	0.247	***	***	0.090	0.041	0.138
	STAT4	0.341	***	***	0.304	***	***
	STAT1	0.352	***	***	0.425	***	***
	IFN-γ (IFNG)	0.228	***	***	0.149	0.001	0.003
	IL12A	0.237	***	***	0.151	0.001	0.002
	IL12B	0.291	***	***	0.264	***	***
Th2	GATA3	0.312	***	***	0.335	***	***
	STAT6	0.245	***	***	0.299	***	***
	STAT5A	0.331	***	***	0.300	***	***
	IL13	− 0.012	0.782	0.845	0.059	0.183	0.519
Treg	FOXP3	0.215	***	***	0.314	***	***
	CCR8	0.336	***	***	0.318	***	***
	STAT5B	0.378	***	***	0.194	***	***
	TGFβ (TGFB1)	0.229	***	***	0.180	***	***

PRAD, prostate adenocarcinoma; THCA, thyroid carcinoma; Th, T helper cell; Treg, regulatory T cell; Cor, R value of Spearman’s correlation; ***P < 0.001

### Enrichment analysis of XRCC4-related proteins and genes

**Fig. 9:**
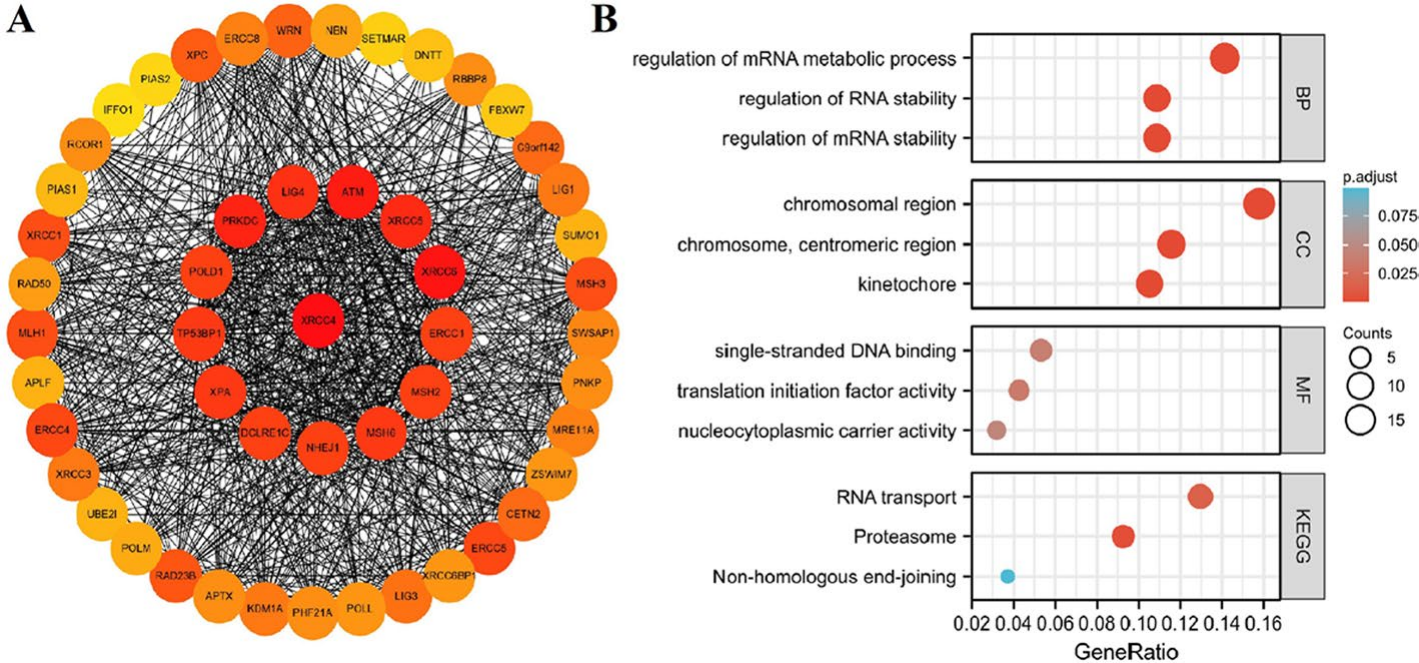
Protein–protein interaction network of XRCC4-binding proteins and XRCC4-related genes enrichment analysis. **A** PPI of XRCC4-binding proteins using the STRING tool; **B** GO and KEGG results of XRCC4-related genes

To further explore the role of XRCC4 in tumorigenesis, we screened out the targeting XRCC4-binding proteins and the XRCC4 expression-related genes for enrichment analyses. Via the STRING database, we obtained 50 XRCC4-binding proteins, and Fig. 9A showed the protein–protein interactions network. Next, we obtained the top 100 XRCC4 expression-related genes via the GEPIA2 database. The main gene ontology (GO) terms of XRCC4 and its co-expression genes are primarily enriched in “regulation of mRNA metabolic process”, “chromosomal region” and “single-stranded DNA binding”. The KEGG pathways enrichment result show that they are primarily involved in “RNA transport”, “proteasome” and “non-homologous” (Fig. 9B).

### Drug sensitivity analysis of XRCC4

**Fig. 10:**
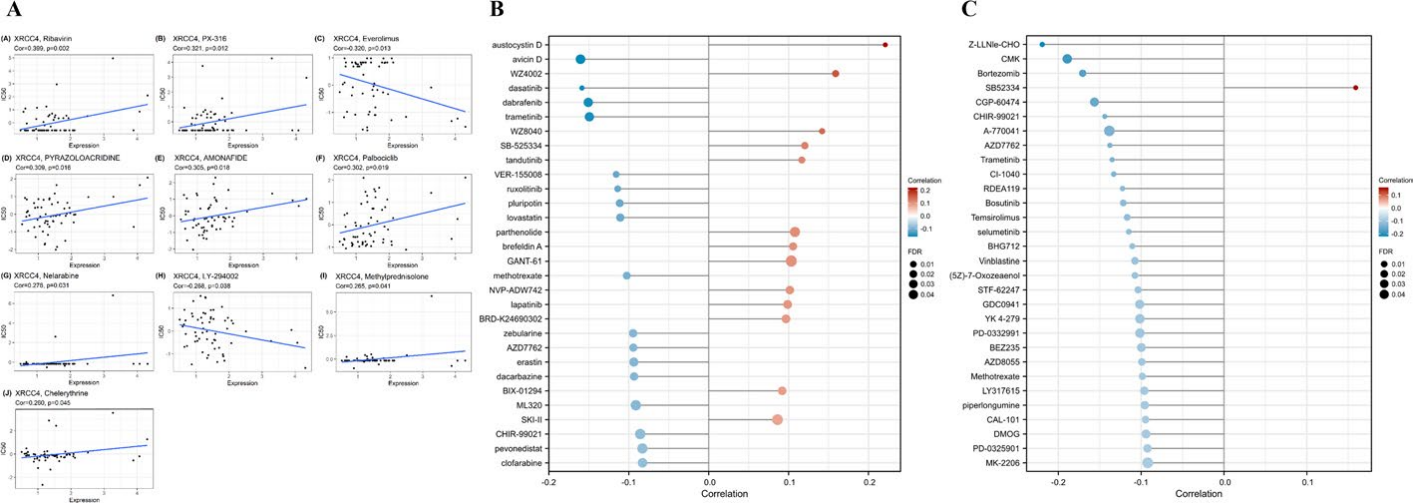
Correlation between XRCC4 expression and the drug sensitivity across cancer types. **A** By CellMiner, **B** By GDSC and **C** by CTRP

The findings presented above suggest that XRCC4 plays a role in the prognosis and immunity of cancers. Therefore, we further analyzed the potential associations between XRCC4 expression and drug sensitivity via three database, including the CellMiner, CTRP and GDSC database. Notably, XRCC4 expression was significantly negative associated with IC50 of Everolimus (*r* = − 0.320, *P* = 0.013) and LY-294002 (*r* = − 0.268, *P* = 0.038), which is equivalent to positive associated with drug sensitivity. The result also showed that XRCC4 expression was significantly positive associated with IC50 of Ribavirin (*r* = 0.399, *P* = 0.002), PX-316 (*r* = 0.321, *P* = 0.012), Pyrazoloacridine (*r* = 0.309, *P* = 0.016), Amonafide (*r* = 0.305, *P* = 0.018), Palbociclib (*r* = 0.302, *P* = 0.019), Nelarabine (*r* = 0.278, *P* = 0.031), Methylprednisolone (*r* = 0.265, *P* = 0.041), and Chelerythrine (*r* = 0.260, *P* = 0.045) (Fig. 10A). To further verify the correlations, we conducted drug sensitivity analysis by using the data accessed from GDSC and CTRP database. As we can see from the Fig. 10B and Fig. 10C, the expression of XRCC4 was negatively correlated with IC50 of four drugs (FDR < 0.1) in GDSC and CTRP simultaneously, including CHIR-99021, AZD7762, Methotrexate, and Trametinib, with correlation of − 0.1439 (*P* < 0.0001), − 0.1378 (*P* = 0.0003), − 0.0985 (*P* = 0.0076) and − 0.1351 (*P* = 0.0002) in GDSC, and − 0.0857 (*P* = 0.0412), − 0.0943 (*P* = 0.0150), − 0.1025 (*P* = 0.0125) and − 0.1493 (*P* = 0.0268), respectively.

## Discussion

In this integrated bioinformatics analysis, we comprehensively investigated the features of XRCC4 in multi-faceted, including expression pattern, prognosis, clinical subtypes, genetic mutation, TME immune cell infiltration, function and signaling pathway and drug sensitivity across cancer types. It was found that XRCC4 is significantly overexpressed in most of the human cancers and negative associated with the prognosis. The relationships between XRCC4 and immune-related genes and TME infiltration immune cells of this study’s result could, to a certain extent, explain the correlations between XRCC4 and patients’ prognosis. The findings provide mechanistic evidence supporting the hypothesis that XRCC4 plays a vital role in the formation, treatment response and survival prognosis across cancer types.

XRCC4 located on chromosome 5q14.2 and consist of 13 exons, plays an important role in the process of repairing DNA double-stranded breaks in the final step of NHEJ by interacting with Ku70/Ku80 complex [7–9, 27]. XRCC4 deficiency was identified as one risk factor for neurological abnormalities [28]. The deficiency of XRCC4 could also associate with short stature, gonadal failure and early-onset metabolic syndrome [29]. Consistent with the findings of the present study, previous researches have confirmed that XRCC4 overexpression in uterine cervical cancer [10], esophageal cancer [12] and hepatocellular carcinoma [13]. It was also reported that abnormal expression of XRCC4 was correlated with chemotherapy and radiotherapy response [10], 12, 13. In addition, we found the mRNA expression of XRCC4 was significantly overexpressed in other 15 of 25 types of human cancers, while was low expressed in KICH and PRAD compared to the normal tissues, the result was validated by the data from the paired comparison via Xiantao Academic. Moreover, the multiple organ tissue arrays obtained from HAP showed the protein expression of XRCC4 was upregulated in LIHC, BRCA, LUAD and Renal-cancer tissues. Previous studies have reported that XRCC4 polymorphisms were related to the risk of prostate cancer [30], lung cancer [31] and bladder cancer [32]. A case–control study carried out among 134 prostate cancer and 134 age-matched healthy controls reported XRCC4 single nucleotide polymorphisms (SNP) increases the risk of susceptibility to prostate cancer [30]. A study among Chinese population showed that SNPs of XRCC4 were a risk factor for non-small-cell lung cancer carcinogenesis [31]. Rama et.al found XRCC4 genotype was associated with urothelial bladder cancer risk [32]. In the present study, we found mutation and deep deletion are the highest XRCC4 alteration in pan-caner. The findings provide new evidence for the potential role of XRCC4 exerted during tumorigenesis process in human cancer.

Although XRCC4 was overexpressed in most cancer types, the result of survival prognosis analysis for the XRCC4 indicated different results for different tumors. We observed that XRCC4 overexpression was associated with poor OS, DFS or RFS in 11 types of cancers. Positive correlations between high expression of XRCC4 and favorable OS, DFS for KIRC, and favorable OS for THYM were also observed. Given the potential impact of XRCC4 on tumorigenesis and prognosis, we further analyzed XRCC4 expression in different immune and molecular subtypes of human pan-cancer to investigate the underlying mechanism. Similar to previous reports, XRCC4 expression was significantly different in different immune and molecular subtypes in most cancers, which might suggest that XRCC4 is a potential diagnostic pan-cancer biomarker that involved in immune regulation [33].

Multiple studies have identified that tumor-infiltrating lymphocytes (TILs) in the TME are the independent predictors for prognosis and immunotherapy of cancer patients [34, 35]. In this work, we found XRCC4 expression was significant correlated with TILs, significant and nonsignificant correlations were found in different cells and cancer types. For instance, XRCC4 expression was significant negative correlated with CD8^+^ T cells infiltration level in LGG, but was significant positive correlated with CD8^+^ T cells infiltration level in THYM and TGCT. The correlations between XRCC4 expression and macrophage infiltration were negative and positive in HNSC-HPV + and LGG, respectively. Noteworthy, XRCC4 expression was significant positive correlated with infiltration levels of CD4^+^ T cells, CD8^+^ T cells, dendritic cells, macrophage, and neutrophil, which infiltrated in TME have been known as both anti- and pro-tumor properties promote immunosuppression in tumor immune escape [36, 37]. XRCC4 expression was also found to be significant associated with 42 of 49 examined marker genes in PRAD, which provides evidence for the potential vital role of XRCC4 played in immunoregulation in PRAD. It is reported that, XRCC4 could potentiate innate immune response [38]. The above results indicated the potential of XRCC4 as a target in anticancer immunotherapy.

According to the results of GO and KEGG analysis, we further validated that XRCC4 and related genes indeed involved in DNA damage response. As discussed above, XRCC4 expression was related to the radiotherapy response of cancer patients, but little was researched in the field of drug sensitivity or resistance till now. From the drug sensitivity analysis using CellMiner™ database, we found that high XRCC4 expression was significantly associated with a better response to Everolimus, an inhibitor of mTOR [39] has been used for treatment of HER2-negative breast cancer, progressive neuroendocrine tumors, advanced RCC and so on [40]. The results indicated that XRCC4 expression level may influence the response to the targeted molecular therapy drugs, and XRCC4 has potential to be a novel target for cancer therapeutics in the future.

There are several limitations in the present study. First, our sequencing and microarray data were obtained from open accessed databases, which inevitably introduced systematic bias caused by the heterogeneity of samples in different studies. Second, the potential influence of XRCC4 on the tumorigenesis should be further clarified based on experiments and large-scale clinical studies. Third, the correlations between XRCC4 expression and prognosis of human cancers and immune cell infiltration lack direct evidence. Fourth, the effect of XRCC4 on targeted therapy response need to be explained and verified by further mechanism studies.

## Conclusions

This comprehensive pan-cancer analysis study elaborated statistical correlations between XRCC4 expression and clinical prognosis, molecular and immune subtypes, immune cell infiltration, immune-related genes and drug sensitivity. Besides, we demonstrated XRCC4 expression characteristic, mutation pattern and function of related-proteins in a variety of human cancers. Although these results should be validated by additional researches, they indicated that XRCC4 may play a role in the prognosis and immunotherapy response in cancers and is a promising therapy target.

## Supplementary information

**Additional file 1. **XRCC4-related cell type distribution using single-cell RNA sequencing database. (**A**, **B**, **D**, **E**, **G**, **H**, **J**, **K**, **M**, **N**, **P**, **Q**) The cell types and their distribution in BRCA_GSE176078, KICH_GSE159115, KIRC_GSE171306, LIHC_GSE146409, PRAD_GSE141445 and STAD_GSE134520 datasets. (**C**, **F**, **I**, **L**, **O**, **R**) Distribution of XRCC4 expression in different cell types using violin plot.

**Additional file 2**. The relationship between XRCC4 expression and pan-cancer immune subtypes. **(A)** in BLCA, **(B) **in SKCM, **(C) **in KIRP, **(D)** in THCA, **(E)** in KIRC, **(F) **in OV, **(G)** in LUSC, **(H)** in SARC and** (I)** in READ.

**Additional file 3**. The relationship between XRCC4 expression and infiltrating immune cells. The correlation was adjusted by tumor purity. **(A)** in BLCA, **(B) **in HNSC and** (C) **in THYM.

**Additional file 4**. Correlations between XRCC4 expression and immune checkpoint genes.

**Additional file 5**. Correlation between XRCC4 expression and immune cells infiltarion in pan-cancer.

**Additional file 6**. Association between XRCC4 expression and marker genes of immune cells.

## Data Availability

The datasets generated and/or analyzed during the current study are publicly available in the TIMER2 database (http://timer.cistrome.org/), GTEx database (https://gtexportal.org/home/), XianTao Academic (https://www.xiantao.love/products), the Kaplan–Meier Plotter platform (www.kmplot.com) and the CellMiner™ (https://discover.nci.nih.gov/cellminer/home.do).
