# Supplementary material for: Systematic analysis identifies XRCC4 as a potential immunological and prognostic biomarker associated with pan-cancer
Source: BMC Bioinformatics. 2023 Feb 10;24:44. doi: 10.1186/s12859-023-05165-8 (PMC9921312; doi:10.1186/s12859-023-05165-8)

Additional File 1

XRCC4-related cell type distribution using single-cell RNA sequencing database. **(A, B, D, E, G, H, J, K, M, N, P, Q)** The cell types and their distribution in BRCA_GSE176078, KICH_GSE159115, KIRC_GSE171306, LIHC_GSE146409, PRAD_GSE141445 and STAD_GSE134520 datasets. **(C, F, I, L, O, R)** Distribution of XRCC4 expression in different cell types using violin plot.
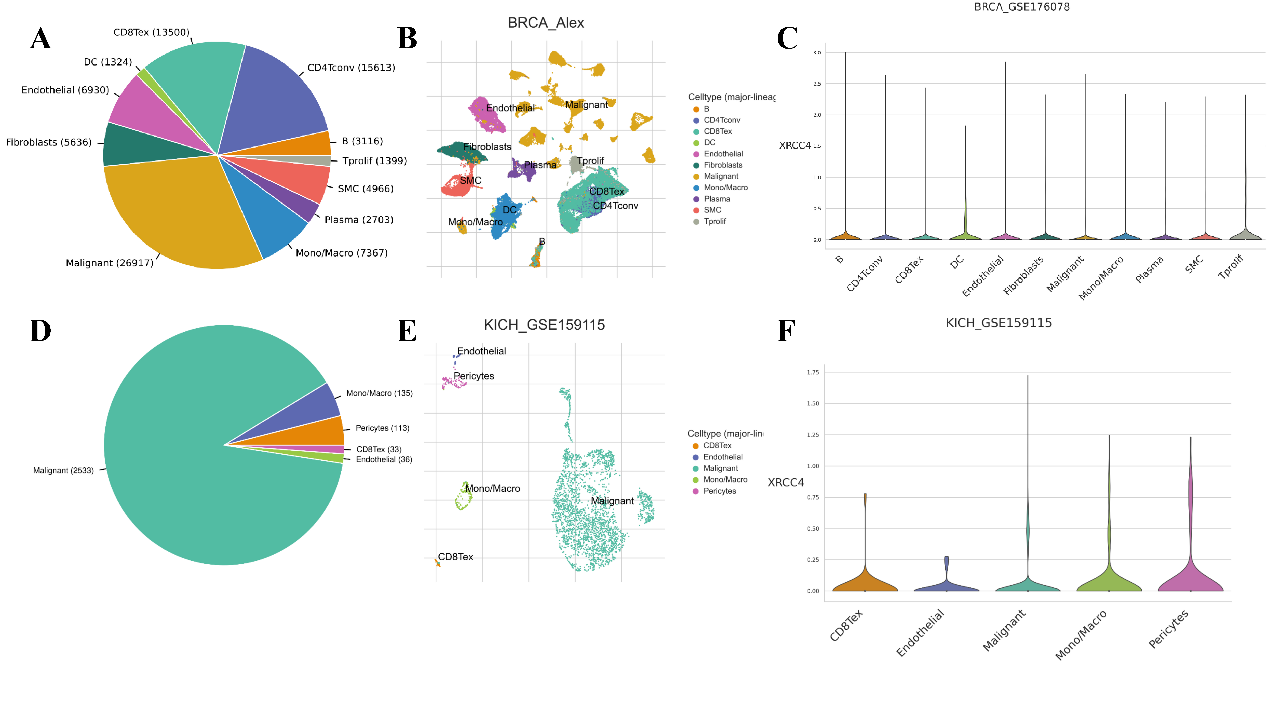


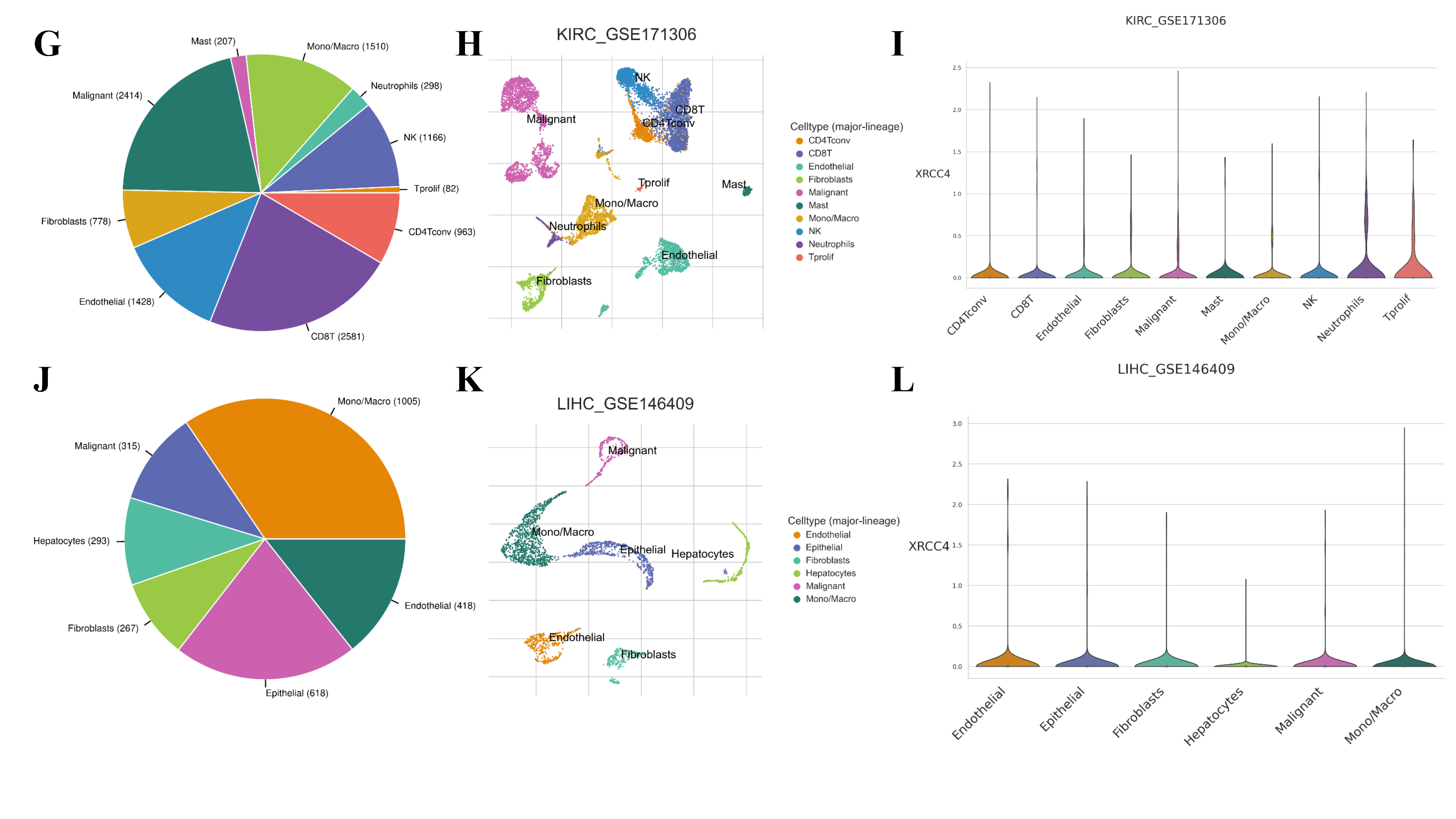

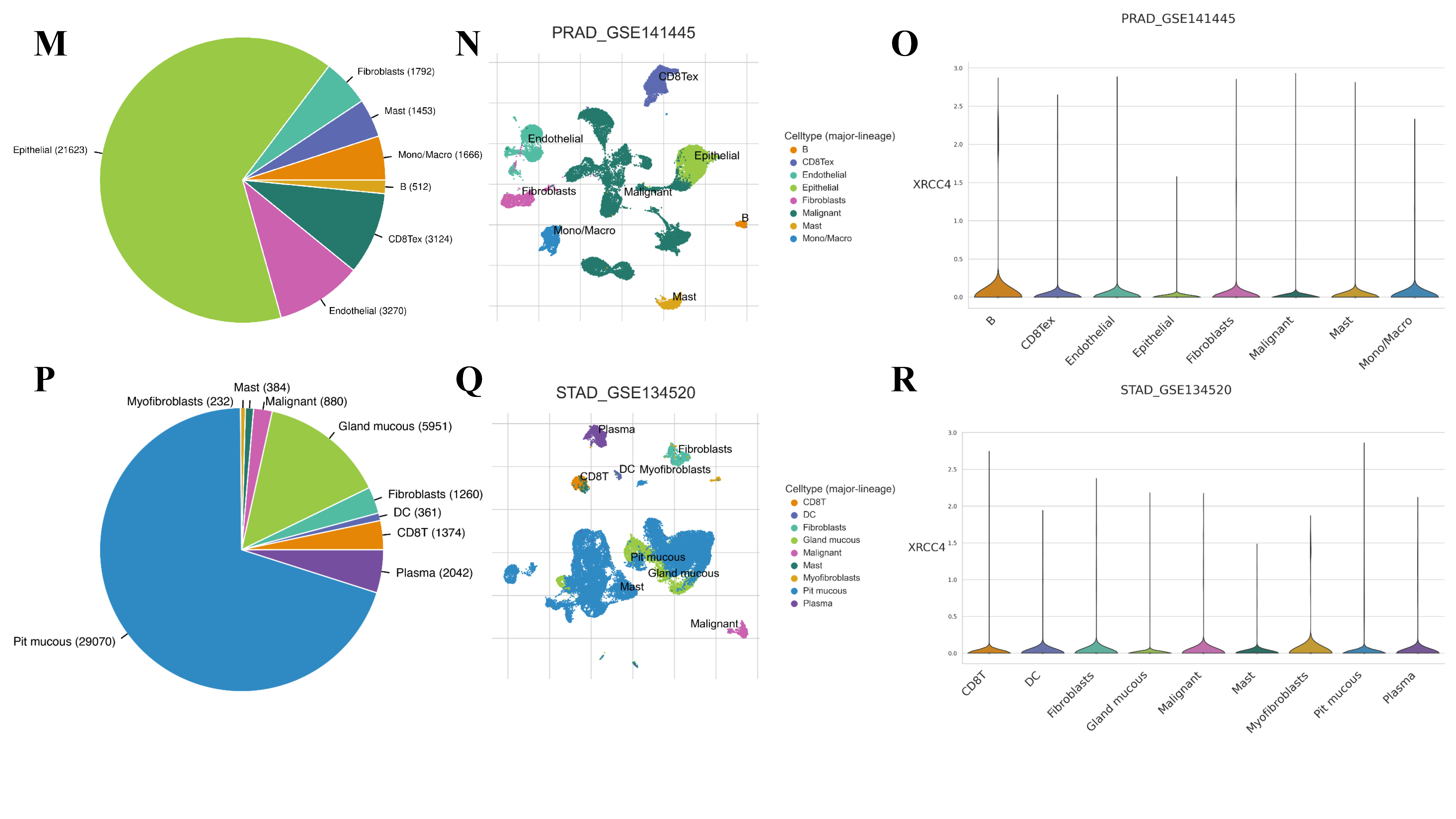

Supplement: Supplementary file 1 — Additional file 1. XRCC4-related cell type distribution using single-cell RNA sequencing database. (A, B, D, E, G, H, J, K, M, N, P, Q) The cell types and their distribution in BRCA_GSE176078, KICH_GSE159115, KIRC_GSE171306, LIHC_GSE146409, PRAD_GSE141445 and STAD_GSE134520 datasets. (C, F, I, L, O, R) Distribution of XRCC4 expression in different cell types using violin plot. [file 12859_2023_5165_MOESM1_ESM.docx]
