# Supplementary material for: Systematic analysis identifies XRCC4 as a potential immunological and prognostic biomarker associated with pan-cancer
Source: BMC Bioinformatics. 2023 Feb 10;24:44. doi: 10.1186/s12859-023-05165-8 (PMC9921312; doi:10.1186/s12859-023-05165-8)

Additional File 2

The relationship between XRCC4 expression and pan-cancer immune subtypes. (A) in BLCA, (B) in SKCM, (C) in KIRP, (D) in THCA, (E) in KIRC, (F) in OV, (G) in LUSC, (H) in SARC and (I) in READ.


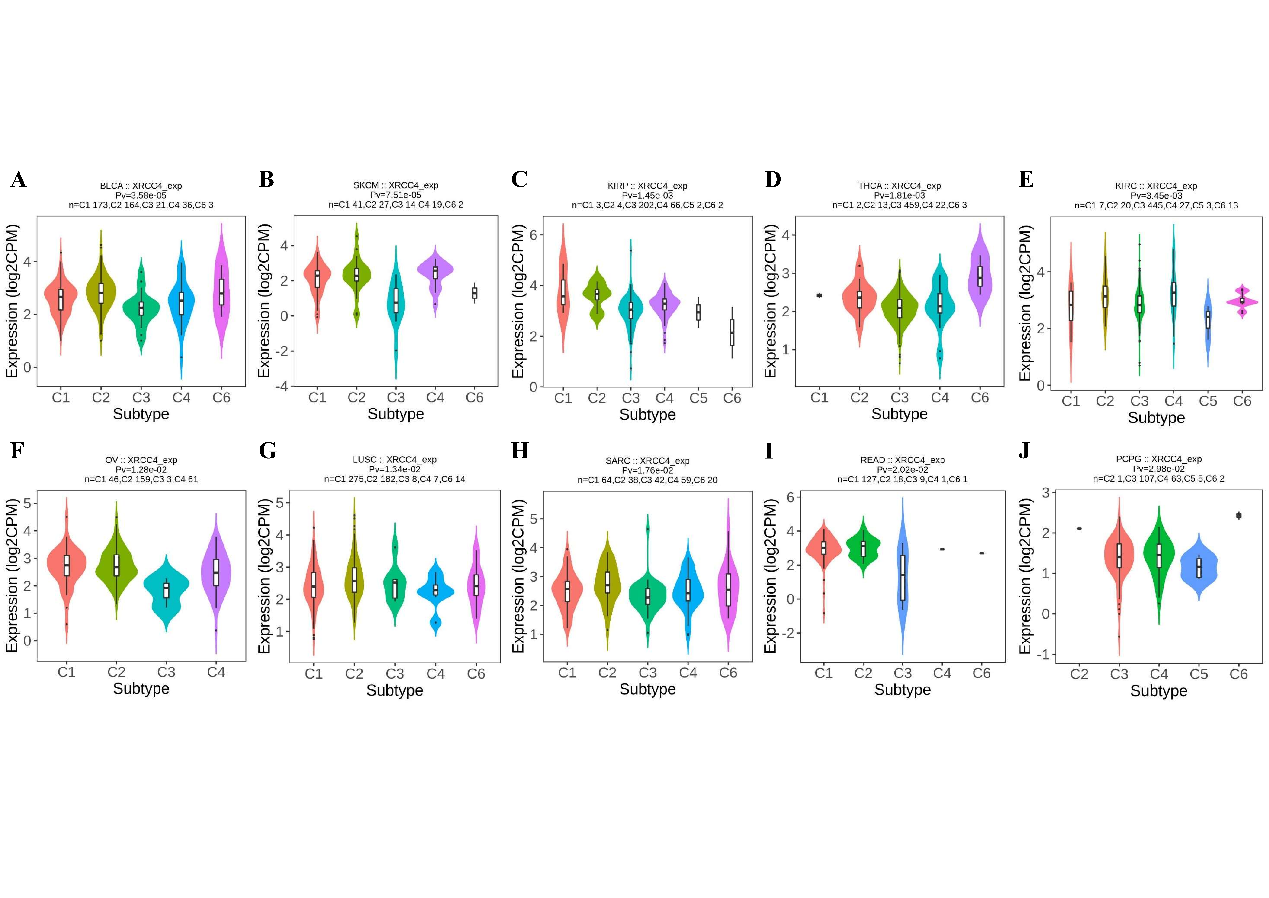

Supplement: Supplementary file 2 — Additional file 2. The relationship between XRCC4 expression and pan-cancer immune subtypes. (A) in BLCA, (B) in SKCM, (C) in KIRP, (D) in THCA, (E) in KIRC, (F) in OV, (G) in LUSC, (H) in SARC and (I) in READ. [file 12859_2023_5165_MOESM2_ESM.docx]
