# Supplementary material for: Systematic analysis identifies XRCC4 as a potential immunological and prognostic biomarker associated with pan-cancer
Source: BMC Bioinformatics. 2023 Feb 10;24:44. doi: 10.1186/s12859-023-05165-8 (PMC9921312; doi:10.1186/s12859-023-05165-8)

Additional File 3

The relationship between XRCC4 expression and infiltrating immune cells. The correlation was adjusted by tumor purity. **(A)** in BLCA, **(B)** in HNSC and **(C)** in THYM.


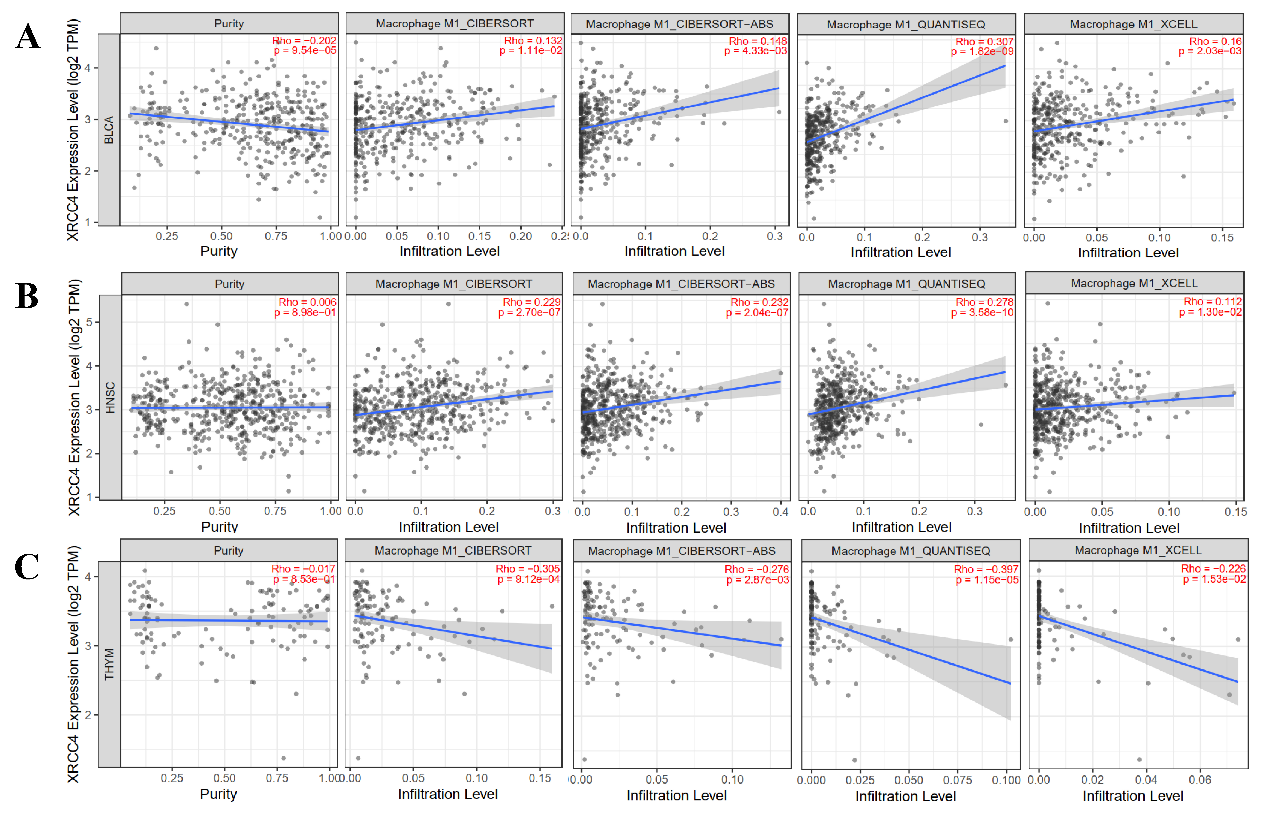

Supplement: Supplementary file 3 — Additional file 3. The relationship between XRCC4 expression and infiltrating immune cells. The correlation was adjusted by tumor purity. (A) in BLCA, (B) in HNSC and (C) in THYM. [file 12859_2023_5165_MOESM3_ESM.docx]
